# Potential risk factors for diabetic neuropathy: a case control study

**DOI:** 10.1186/1471-2377-5-24

**Published:** 2005-12-10

**Authors:** Fargol Booya, Fatemeh Bandarian, Bagher Larijani, Mohammad Pajouhi, Mahdi Nooraei, Jamshid Lotfi

**Affiliations:** 1 Researcher, Endocrinology and Metabolism Research Center (EMRC), Tehran University of Medical Sciences, Tehran, Iran; 2 Professor of Internal Medicine, Endocrinology, Endocrinology and Metabolism Research Center, Tehran University of Medical Sciences, Tehran, Iran; 3 Epidemiologist, Endocrinology and Metabolism Research Center, Tehran University of Medical Sciences, Tehran, Iran; 4 Neurologist, Department of Neurology, Shariati hospital, Tehran University of Medical Sciences, Tehran, Iran

## Abstract

**Background:**

Diabetes mellitus type II afflicts at least 2 million people in Iran. Neuropathy is one of the most common complications of diabetes and lowers the patient's quality of life. Since neuropathy often leads to ulceration and amputation, we have tried to elucidate the factors that can affect its progression.

**Methods:**

In this case-control study, 110 diabetic patients were selected from the Shariati Hospital diabetes clinic. Michigan Neuropathic Diabetic Scoring (MNDS) was used to differentiate cases from controls. The diagnosis of neuropathy was confirmed by nerve conduction studies (nerve conduction velocity and electromyography). The multiple factors compared between the two groups included consumption of angiotensin converting enzyme inhibitors (ACEI), blood pressure, serum lipid level, sex, smoking, method of diabetes control and its quality.

**Results:**

Statistically significant relationships were found between neuropathy and age, gender, quality of diabetes control and duration of disease (P values in the order: 0.04, 0.04, < 0.001 and 0.005). No correlation was found with any atherosclerosis risk factor (high BP, hyperlipidemia, cigarette smoking).

**Conclusion:**

In this study, hyperglycemia was the only modifiable risk factor for diabetic neuropathy. Glycemic control reduces the incidence of neuropathy, slows its progression and improves the diabetic patient's quality of life. More attention must be paid to elderly male diabetic patients with poor diabetes control with regard to regular foot examinations and more practical education.

## Background

Diabetes mellitus (DM) is one of the most widespread chronic diseases in the world. Nearly 7.5% of Iranian people are affected by DM type II [1]. DM has two types of complications: microvascular and macrovascular. One of the most frequently-occurring microvascular complications is diabetic neuropathy (DN), of which the most common type is distal symmetrical neuropathy or polyneuropathy. This results in significant disability and morbidity [2,3]. Complications of DN include severe pain, loss of ambulation and increased risk of foot ulceration and amputation.

Incidences of polyneuropathy have been reported in 10–50% of patients with diabetes [3]. At the time of diagnosis, neuropathy is present in 10% of diabetic patients and overall in 50% of patients with a 25-year history of the disease [4,5]. Life-time risk of foot amputation is 15% in patients with diabetic polyneuropathy [5]. Polyneuropathy is the first step in the generation of diabetic foot ulcer. It produces an anesthetic foot defective in proprioception and therefore exposed to inappropriate loading. Foot ulcers develop in risk areas that are exact pressure points [6].

Different hypotheses have been proposed to explain the various modes of progression of DN. It has been suggested that consumption of oral hypoglycemic agents such as glyburide [7] and angiotensin converting enzyme inhibitors (ACEI) inhibit the progression of neuropathy irrespective of blood glucose level [8-10]. Atherosclerosis risk factors are thought to promote DN [6]. The induction of mononeuropathy is closely associated with high blood pressure (BP), hyperlipidemia and cigarette smoking [6]. Since neuropathy can lead to ulceration and amputation, we have tried to assess the relationships between these risk factors and sensory/motor polyneuropathy. Early diagnosis and treatment of DN is important for preventing secondary complications and improving quality of life.

## Methods

One hundred and ten diabetic patients participated in this case-control study (55 patients in each group). Controls and cases were chosen from the Shariati Hospital diabetes outpatient clinic by simple randomized sampling. A control subject was a diabetic patient with no evidence of DN, and a case was a diabetic patient with neuropathy. The patients' ages ranged between 20 and 80 years. Exclusion criteria were creatinine >2 mg/dl, specific neurological disease (M.S, stroke, etc.), other causes of neuropathy (B_12 _deficiency, alcoholism, etc.), loss of dorsalis pedis pulses and less than 5 years duration of disease. Informed consent was completed by all participants before they were enrolled in the study. The study design was approved by the research ethics committee of the Endocrinology and Metabolism Research Center of Tehran University of Medical Sciences.

In order to differentiate between cases and controls, Michigan Neuropathic Diabetic Scoring (MNDS) was used [11]. This system gives a score in the range 0–8, based on evaluation of 4 different factors in the each leg. These factors are: appearance of foot (dry skin, callus, deformities, fissure, and infection), presence of ulcer, Achilles tendon reflex and vibration perception in the great toe (measured with a 128 Hz tuning fork). Each component may be given a score of 0.5 or 1 on the basis of the relevant signs. This scoring system has sensitivity and specificity of nearly 95% [12]. A neuropathic foot usually scores 3 or higher, a normal foot 2.5 or lower. Cases with a diagnosis of neuropathy according to the Michigan scoring system were confirmed by nerve conduction studies (EMG-NCV). For this purpose, nerve conduction velocity, amplitude, duration and latency were assessed in 5 sensory/motor nerves (Median, Ulnar, Tibial, Proneal and Sural) on the non-dominant side of the body. At least one abnormal test in more than one of these nerves was considered indicative of neuropathy [11].

Detailed information on each patient's age, sex, type and duration of diabetes mellitus, mode of treatment (insulin, oral hypoglycemic agents or both), degree of blood glucose control (bad, fair, good), presence of hypertension, hyperlipidemia (serum total cholesterol level), ACEI consumption and smoking was recorded and compared between cases and controls.

The quality of diabetes control was classified according to the average glycosylated hemoglobin (HbA_1C_) over the previous year. Average HbA_1C _≤7.5 was considered good quality control; average 7.6< HbA_1C _≤9 was considered fair control, and average HbA_1C _≥9.1 poor control. All necessary data were retrieved from the patient records in the diabetes outpatient clinic of Shariati Hospital. Patients with BP≥140/90 were considered hypertensive. Patients with total cholesterol ≥250 were considered hyperlipidemic. HbA_1C _was measured by HPLC. The total cholesterol level was measured by calorimetry (Pars Azmoon kit), and fasting blood sugar (FBS) by the glucose oxidase method (Pars Azmoon kit).

SPSS 10 software was used for data entry and analysis. Since multiple factors were analyzed, multivariate analysis was used. Logistic regression was the appropriate mode for analyzing the multiple risk factors in cases and controls

## Results

Of the 110 patients, 78% (79) were female and 22% (31) were male. The mean age was 55.1 ± 13.2 (20–80 years). All but one of the patients had type II DM. Mean fasting blood glucose and average duration of disease in the study population were 140.5 ± 8 mg/dl and 12.9 ± 7 years, respectively. Table 1 shows age, duration of disease, mean FBS, mean 2 hour post-parendial blood glucose (BS2hpp), HbA_1C _and total cholesterol in cases and controls. Table 2 shows the frequency of potential risk factors for polyneuropathy in the study population. No significant relationships were found between distal symmetric sensory/motor polyneuropathy and cigarette smoking, ACEI consumption, BP or cholesterol level.

**Table 1:** Comparison of variables between patients with and without diabetes neuropathy

**Variables**	**Patients with neuropathy**	**Patients without neuropathy**	**p-value**
**Age (year)**	58.4 ± 10.5	55 ± 10.7	0.04
**Sex**	61.8% F, 38.2% M	81.8% F, 18.2% M	0.02
**FBS (mg/ml)**	143.6 ± 60.2	130.8 ± 63.5	0.13
**BS2hpp (mg/ml)**	252 ± 82	234 ± 86	0.09
**HbA_1C_(%)**	8.2 ± 2.5	7.9 ± 2.7	0.42
**Total cholesterol (mg/dl)**	214.9 ± 26.4	193.3 ± 29.3	0.001
**Duration of disease (year)**	14.2 ± 7.4	11.6 ± 9.4	0.03
**MNDs Score**	5.5 ± 1.4	1.1 ± 5.9	0.0001

**Table 2:** Frequency of potential diabetic neuropathy risk factors in 110 diabetic patients

**Variables**		**Prevalence (%)**
**Hypertension**		41.8
**ACEI usage**		28.2
**HbA_1C_**		
	**Poor control**	45.5
	**Faire control**	18.2
	**Good control**	36.3
**Cigarette smoking**		
	**None**	78.2
	**More than 6 months of withdraw**	14.5
	**less than 10 cigarette/day**	7.3
**Hypercholesterolemia**		42.7

**Figure 1:**
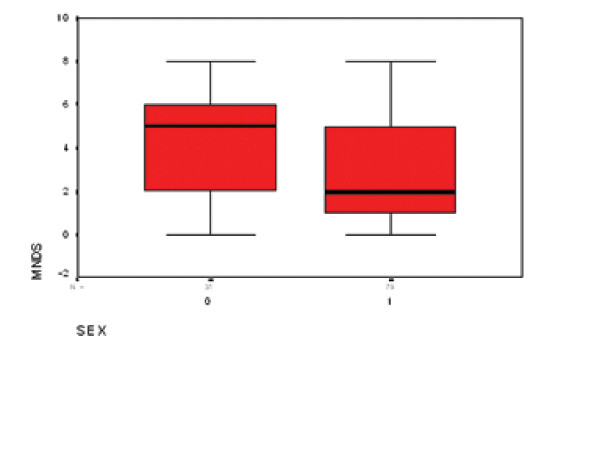
Box plot demonstrating relationship between MNDS score and sex (0 = male, 1 = female). The dark line in the plot is equivalent to mean MNDS score. Average score is higher in males.

Multivariate analysis revealed statistically significant relationships between DN and age, gender, degree of diabetes control and duration of disease (P values: 0.04, 0.04, < 0.001 and 0.005, respectively). Neuropathy was more frequent in men than women (odds ratio male/female 2.9, figure 1). The multivariate analysis results are shown in Table 3.

**Table 3:** The association of sex, duration of disease and quality of diabetes control with diabetic neuropathy (result of multivariate analysis, logistic regression)

**Variable**	**β**	**P-value**	**Odds ratio**
**Sex (male/female)**	1.56	0.04	2.9
**Duration of disease**	5.1	0.005	1.1
**Quality of diabetes control (fair/bad)**	-2.2	<0.001	0.2
**Quality of diabetes control (good/bad)**	-1.2	0.04	0.3
**Constant**	-0.54	0.29	1.7

**Figure 2:**
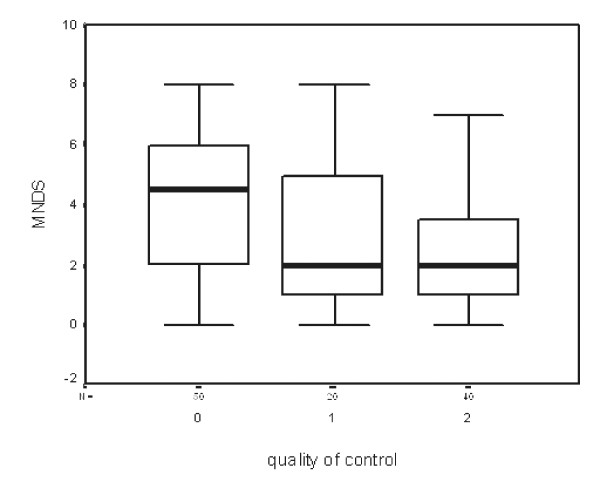
Box plot demonstrating relationship between MNDS score and quality of diabetes control (0 = poor, 1 = fair, 2 = good). The dark line in the plot is equivalent to mean MNDS score. Average score is higher in poor control of diabetes.

Poor diabetes control increases the likelihood of neuropathy 0.3 times (odds ratio good control/bad control 0.3 and fair/bad 0.2). Figure 2 shows the relationship between quality of diabetes control (good, fair and bad) and MNDS score. Each additional year of disease increases the likelihood of neuropathy 1.1-fold.

## Discussion

Diabetic polyneuropathy is a common complication of DM with high morbidity and impairment of quality of life. Tesfye et al. [13] studied 3,250 diabetic patients and reported an overall prevalence of peripheral neuropathy in 28% of them. The condition was significantly associated with age, duration of disease, height, diastolic blood pressure, smoking status, low HDL cholesterol level, high triglyceride level and HbA_1C_.

The Ashok study [14] showed significant relationships only with age and duration of disease. No other association was detected. Other studies have shown associations of neuropathy with age [14-19], duration of disease [14-20], metabolic control [15,18-21], height [15,22,23], cigarette smoking [15,19,24], retinopathy [15,21] and reduced HDL level [15]. The results of the present study confirm previous reports regarding the association of neuropathy with male gender, age, glycemic control (HbA_1C_) and duration of disease. Our data are also concordant with the DCCT (Diabetes Control and Complications Trial) [25] and UKPDS (United Kingdom Prospective Diabetes Study) results [26], which used EMG-NCV to identify neuropathic patients. Our finding that male gender is associated with neuropathy is consistent with the DCCT report [25]. Therefore, it can be concluded that MNDS criteria can be used with high confidence as an outpatient screening method. In our study, no statistically significant relationship was found between peripheral neuropathy and ACEI or consumption of oral hypoglycemic agents. Polyneuropathy was not significantly related to BP, smoking or hyperlipidemia. Most of our patients were nonsmokers, so it was impossible to examine the possible association between smoking and neuropathy critically.

Further studies using a randomized clinical trial are needed to evaluate the effects of ACEI and oral hypoglycemic agents on neuropathy.

## Conclusion

Since hyperglycemia is a modifiable risk factor for diabetic neuropathy, intensive glycemic control is the most effective established therapy for reducing the incidence or slowing the progression of neuropathy and improving quality of life in diabetic patients. According to the results of the present study, better care should be given to elderly male diabetic patients with poor diabetic control in terms of regular foot examinations and more practical education.

## Competing interests

The author(s) declare that they have no competing interests.

## Authors' contributions

FB and FB: drafted the manuscript and coordinated the study

BL, MP, JL: conceived of the study and participated in the design of the study

MN: performed statistical analysis

All authors read and approved the final manuscript

## Pre-publication history

The pre-publication history for this paper can be accessed here:


